# Machine Learning Prediction of Foodborne Disease Pathogens: Algorithm Development and Validation Study

**DOI:** 10.2196/24924

**Published:** 2021-01-26

**Authors:** Hanxue Wang, Wenjuan Cui, Yunchang Guo, Yi Du, Yuanchun Zhou

**Affiliations:** 1 Computer Network Information Center Chinese Academy of Sciences Beijing China; 2 Chinese Academy of Sciences University Beijing China; 3 China National Center for Food Safety Risk Assessment Beijing China

**Keywords:** foodborne disease, pathogens prediction, machine learning

## Abstract

**Background:**

Foodborne diseases, as a type of disease with a high global incidence, place a heavy burden on public health and social economy. Foodborne pathogens, as the main factor of foodborne diseases, play an important role in the treatment and prevention of foodborne diseases; however, foodborne diseases caused by different pathogens lack specificity in clinical features, and there is a low proportion of clinically actual pathogen detection in real life.

**Objective:**

We aimed to analyze foodborne disease case data, select appropriate features based on analysis results, and use machine learning methods to classify foodborne disease pathogens to predict foodborne disease pathogens that have not been tested.

**Methods:**

We extracted features such as space, time, and exposed food from foodborne disease case data and analyzed the relationship between these features and the foodborne disease pathogens using a variety of machine learning methods to classify foodborne disease pathogens. We compared the results of 4 models to obtain the pathogen prediction model with the highest accuracy.

**Results:**

The gradient boost decision tree model obtained the highest accuracy, with accuracy approaching 69% in identifying 4 pathogens including Salmonella, Norovirus, Escherichia coli, and Vibrio parahaemolyticus. By evaluating the importance of features such as time of illness, geographical longitude and latitude, and diarrhea frequency, we found that they play important roles in classifying the foodborne disease pathogens.

**Conclusions:**

Data analysis can reflect the distribution of some features of foodborne diseases and the relationship among the features. The classification of pathogens based on the analysis results and machine learning methods can provide beneficial support for clinical auxiliary diagnosis and treatment of foodborne diseases.

## Introduction

### Background

Foodborne diseases refer to diseases caused by pathogenic factors such as harmful substances that enter the body through food intake [1]. They are usually associated with contaminated foods and pathogens or viruses contained in foods. A foodborne disease outbreak is defined as an incident in which 2 or more people experience similar diseases after consuming the same food [2]. According to a World Health Organization (WHO) report [3], 600 million people worldwide suffered from diseases caused by eating contaminated food every year, of whom 4.2 million die. According to the Centers for Disease Control (CDC), 48 million people are infected with foodborne diseases every year in the United States, 128,000 of whom are hospitalized and 3000 of whom die [3]. In recent years, China has also begun monitoring foodborne diseases. In 2008, 294,000 people suffered from foodborne diseases, 50,000 of whom were hospitalized and 6 died [4]. Currently, the incidence of foodborne diseases is among the highest in all kinds of diseases [5]. Frequent occurrences of foodborne diseases at home and abroad seriously endanger public health and social economy and have become an important public health and food safety issue in the world. Foodborne disease–related research and prevention efforts are urgent.

Therefore, many researchers at home and abroad study foodborne diseases, including monitoring, identification and outbreak prediction. The Foodborne Diseases Active Surveillance Network was established in the United States to monitor, track, analyze, and prevent foodborne diseases [6]. In recent years, China has also established surveillance platforms for foodborne diseases, such as the National Foodborne Disease Surveillance Reporting System [7], which classifies, stores, monitors, and statistically analyzes foodborne disease surveillance data collected nationwide. Methods for identification and diagnosis of foodborne diseases are mainly categorized into 2 types—one analyzes the molecular subtypes of pathogens using biochemical tests to diagnose foodborne diseases, another often uses statistical analysis or machine learning algorithms to identify disease information that may be included in the data [8]. For foodborne disease outbreak prediction, regression, clustering, hidden Markov model, and some timeseries prediction methods are usually used.

The main cause of foodborne diseases is that patients are infected with contaminated foods, which causes the pathogens to enter the body [9]. Therefore, research on pathogens of foodborne diseases are of great significance. However, the clinical features of foodborne diseases caused by different pathogens are not specific, and it is difficult to intuitively identify pathogens according to patient information and disease description. Traditional pathogen identification methods based on laboratory testing usually take a long time [10]. In recent years, researchers have proposed some methods for rapid detection of pathogens in foodborne diseases [11-13], including nucleic acid, immune, and biosensor methods; however, these methods require very professional equipment, and there are still some limitations in practical applications. Therefore, only a small proportion of foodborne diseases have been carried out the identification of pathogens, which greatly hinders the diagnosis of foodborne diseases and may affect doctors' ability to treat diseases caused by different pathogens and may even result in misdiagnosis. At the same time, the low proportion of foodborne pathogens identification also leads to incomplete disease data for analysis, which has a negative effect on disease burden estimation and outbreak prediction [14].

### Related Work

#### Foodborne Disease Analysis Based on Surveillance Platform Data

The international community has always attached great importance to the research on foodborne diseases and has carried out many related works. The data sources for these studies include surveillance platforms, social networks, hotlines, search engines, and food samplings [15-18]; however, compared with other data sources, the data from surveillance platforms are reliable and authoritative, and the analysis results based on these data are more credible. That is because these data are usually from hospitals or health departments, and the data are all confirmed foodborne disease cases. Therefore, many foodborne disease–related surveillance platforms have been established internationally to support foodborne disease research. In 1995, the United States established the Foodborne Diseases Active Surveillance Network to monitor and track foodborne diseases [6]. The Foodborne Disease Outbreak Surveillance System is a CDC-sponsored platform for collecting information on foodborne disease outbreaks. It collects information on foodborne disease outbreaks into reports and uploads them to National Outbreak Reporting System every year [19,20]. In 2000, WHO established the Global Foodborne Infection Network for the monitoring, control and prevention of foodborne diseases. In addition, there are some other foodborne disease surveillance platforms, such as PulseNet [21] and GenomeTrakr [22]. In recent years, China has also paid attention to the surveillance of foodborne diseases. China Food Safety Risk Assessment Center established a National Foodborne Disease Surveillance Reporting System [7] to collect, store, analyze and track foodborne disease data nationwide. The data in the system contain disease case information, test information, exposed food information, and report information, which can be used for analysis and research on foodborne diseases.

These foodborne disease surveillance platforms provide a unified and authoritative source for foodborne disease data. Research on foodborne diseases using data from surveillance platforms have been popular for a long time [4,23-28]. However, most of foodborne disease research based on surveillance platform data are concentrated on statistical analysis; only a few use the data for disease aggregation analysis and outbreak prediction [29], and it has not yet been proposed to identify pathogens using surveillance platform data. As the traditional methods of pathogens’ identification using biochemical testing are time-consuming and require technical support, a large proportion of the confirmed foodborne disease cases in the surveillance system have not been tested for pathogens, which will affect the subsequent estimation of foodborne disease burden and foodborne disease outbreak prediction [14]. Therefore, an accurate identification approach for foodborne pathogens based on surveillance platform data is still necessary.

#### Foodborne Disease Analysis Based on Machine Learning

Machine learning addresses the question of how to build computers that improve automatically through experience; it is one of the most rapidly growing technical fields [30]. In recent years, machine learning has been widely used in various fields, including epidemiology. Researchers propose many methods based on machine learning to diagnose diseases, predict outbreak of diseases, analyze gene of disease pathogens, and so on [31,32]. The successful application of machine learning in epidemiology has brought enlightenment to the study of foodborne diseases; many works have been carried out to solve foodborne disease problems using machine learning methods. In the identification of foodborne diseases, many studies choose supervised classification models as well as unsupervised clustering methods instead of traditional statistical methods [8], and it is proved that these studies can obtain good results. In the foodborne disease outbreak prediction, researchers also use machine learning methods, such as hidden Markov models [33] and DBScan models [29]. In addition, there are some works using machine learning methods to analyze foodborne pathogens. Several classification models have identified pathogens by using near infrared laser scatter images [13]. Machine learning is applied in the gene sequence analysis of foodborne pathogens, resulting in more accurate and quicker analysis [34]. The decision tree method is also used to mine the association between food, location, and pathogens based on CDC data [35].

Compared with traditional statistical analysis methods, machine learning methods can achieve more accurate result faster and can handle larger and more complex data. Therefore, machine learning methods have become popular methods to solve problems of foodborne diseases. However, most of these studies focus on the identification or prediction of diseases [8,29,31-33], and only a small part of them were carried out for the analysis of disease pathogens [13,34,35]. Often, molecular typing or gene sequence of pathogens rather than disease case information are used. There are a few machine learning–related works proposed to analyze the relationship between pathogens and disease case data from surveillance platform.

## Methods

### Data Description

Our data source was the National Foodborne Disease Surveillance Reporting System [7], which collected 2.6 million foodborne disease cases from 2011 to 2018. About 60,000 of them have been tested and certain pathogens have been identified, accounting for only 3% of all cases. Among the 60,000 tested cases, a total of 26 pathogens were identified, as shown in Table 1. Among them, the China Food Safety Risk Assessment Center focuses on the detection of *Salmonella*, *Norovirus*, *Escherichia coli*, *Vibrio parahaemolyticus*, and *Shigella*, and the first 4 pathogens (*Salmonella*: 26.5%; *Norovirus*: 25.9%; *E coli*: 20.9%; *V parahaemolyticus*: 18.6%) total more than 50,000, accounting for 92% of the total cases, as shown in Table 1. Therefore, in the following work, we mainly focus on these 4 pathogens.

One case data entry contains information on the patient’s age, gender, home address, time of illness, time of treatment, symptoms, diagnosis, and related food information (including food name, food type name, food processing type, food purchase location, and food intake location). There are also samples and sample test items related to the case, including type, number, number of strains, test method, test item category, test item name, and test result. We used pathogen types as labels. In the process of feature selection, we excluded some food and laboratory testing information. As a result, the selected features included patient's age, patient's gender, home address, time of illness, symptoms, diagnosis, food name, and food type.

We conducted exploratory data analysis to understand the feature distribution and guide data preprocessing in the subsequent step. We use the map to show the geographical distribution of the detection rate of the 4 pathogens. Some research indicated that foodborne diseases have a seasonal pattern and that climatic temperature could be a factor of incidence [36]. Therefore, we performed a visual analysis of the detection rate of the 4 pathogens by time. We also calculate the distribution of patients’ age with different pathogens and visualize the distribution of patients’ age. Besides, we also performed a visual analysis of the gender of the patient and the type of exposed food. The food names, symptoms, and diagnosis were textual information; therefore, they were not explored.

**Table 1:** Distribution of pathogens involved in the cases.

Pathogen	Count, n
*Salmonella*	16378
*Norovirus*	16052
*Escherichia coli*	12947
*Vibrio parahaemolyticus*	11503
*Shigella*	2004
*Rotavirus*	1174
*Campylobacteria*	452
Other pathogens	618
*Staphylococcus aureus*	348
Adenovirus	114
*Aeromonas hydrophila*	114
Star shaped virus	112
*Listeria monocytogenes*	97
Zagreb as viruses	75
*Vibrio cholerae*	37
*Vibrio vulnificus*	22
*Yersinia enterocolitica*	17
*Bacillus cereus*	14
Organophosphorus	10
*Enterobacter sakazakii*	7
*E coli* O157: H7/NM	7
Other viruses	5
Coliform count	3
Mold count	2
Hemolytic streptococcus	2
*Clostridium botulinum*	1
Rodenticide class	1
Determination of total number of colonies	1

### Data Preprocessing

The original data formats are described in Table 2. We mapped the 4 pathogens (*Salmonella*, *Norovirus*, *E coli*, and *V parahaemolyticus*) into 4 classification labels. We converted the gender data in nominal format into a binary variable, and extracted the month value from the time of illness as a time attribute. For the age attribute, we used 10-year intervals. Home address is a distinguishable attribute, but it was stored in 3 fields (province, city and district) in the database, and each field was in numeric format. We remapped the 3 fields into text formats according to dictionaries, combined them, and calculated corresponding latitude and longitude as location attributes.

**Table 2:** The original format and description of attribute data.

Attribute	Format	Description
Pathogen name	Nominal	The name of pathogens
Age	Numeric	The age of patients
Gender	Nominal	The gender of patients
Sick time	Date	The time of illness
Province	Numeric	The value of province in patients’ home address after dictionary mapping
City	Numeric	The value of city in patients’ home address after dictionary mapping
District	Numeric	The value of district in patients’ home address after dictionary mapping
Symptom	Text	The symptom information of patients
Diagnosis	Text	The diagnosis information of patients
Food name	Text	The name of food which patients ate
Food type name	Nominal	The type of food which patients ate

Symptom and diagnosis fields were in text format. Each symptom field (or diagnosis field) contained a series of symptoms (or diagnoses), separated by a comma. When we processed the symptom field, word segmentation into a set of symptoms was performed. For the diarrhea symptom, we mapped all diarrhea features that appear in the data to a dictionary. The diarrhea trait of each disease case was expressed as its corresponding value in the dictionary, the diarrhea frequency of each disease case was the value extracted from the disease case, and the diarrhea frequency of cases without diarrhea was expressed as 0. For the vomiting symptom, we selected vomiting frequency as the attribute, and the value was in numeric format. For cases without vomiting, the frequency of vomiting was 0. For the fever symptom, we extracted the body temperature of each disease case and divided the body temperature into 4 grades (no fever, low, medium, high). For other symptoms, we converted them into a collection of binary variables, and we set a threshold to filter out the symptoms that occur too few times. Examples of symptoms after cleaning and transforming are shown in Table 3. For the diagnosis field, we conducted word segmentation and mapped the segmented diagnose into a collection of binary variables.

**Table 3:** Representation of symptoms of example cases.

Symptom field	Vector	
	Example case 1	Example case 2
Diarrhea traits	1	0
Diarrhea frequency	5	0
Fever	0	1
Sick	1	0
Hypourocrinia	1	0
Vomiting frequency	0	3
Thirst	0	0
Weak	0	0
Stomachache	0	0
Pale complexion	0	0
Tenesmus	0	0
Dehydration	0	0

**Figure 1:**
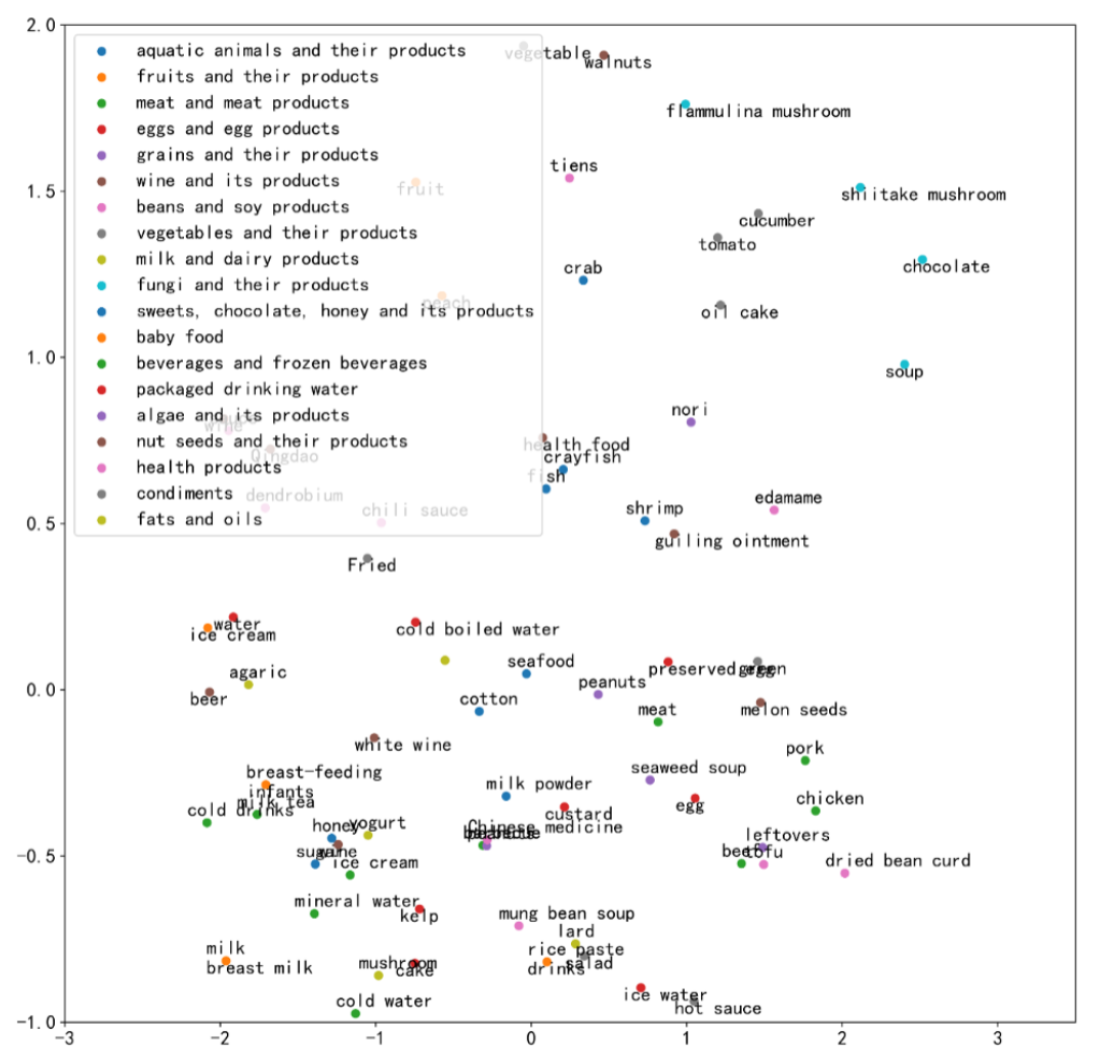
Representation of word vectors of food names in 2 dimensions.

The exposed food information related to the disease case included the type and name of the food. There were 23 food categories which were expressed in nominal format. We converted these into one-hot representations. We first performed data cleaning and word segmentation on the food name field. We removed punctuation, special characters, and numbers, then used the word segmentation tool to segment the food name into a collection of words. Since food name was a text field, we used word2vec, an approach that trains an N-gram language model using a neural network and finds vectors corresponding to the words to learn high quality spatial representation of words from a large amount of unstructured text data [37], to embed food name information into vectors, using an open pretrained Chinese word embedding model [38] to represent the food name that trains text data from Baidu Encyclopedia. After mapping words into vectors, semantically similar words were relatively close in the vector space. To maintain the same dimension in each disease case, we calculated the average value of word vectors for each food name and obtained a 300-dimension vector for each food name field. Then, using variance for feature selection, we determined the final variance threshold and the dimension of the word vectors by comparing the model results under different thresholds to reduce the dimension of word vectors to control the feature dimension within a reasonable range and reduce the training time of model. In addition, we used *t*-distributed stochastic neighbor embedding to reduce the word vectors to 2 dimensions and used a scatter plot to represent word vectors of the top 5 foods (we removed unknown foods, mixed foods, multiple foods and other foods) with the highest frequency among the 19 types [39], shown in Figure 1. Finally, all features were combined into 349-dimension vectors.

### Classification Methods

Statistical analysis revealed the distribution of the 4 pathogens was relatively balanced; therefore, no extra sampling was required. We trained decision tree, random forest, gradient boost decision tree (GBDT), and adaptive boosting models with the processed data in Python (version 3.7; Scikit-learn package [40]) and compared the results to obtain the best classification model.

Decision tree [41] is a nonparametric supervised learning method widely used in classification and regression. It differs from other classifiers that put all the features into the classifier at once. It decomposes the complex decision-making process into recursive steps, dividing the features. It does not require data normalization and has good interpretability [41].

Random forest is an ensemble model based on decision trees that can solve the problem of weak generalizability of decision trees [42]. It builds multiple decision trees and uses voting methods to obtain the final result. Each tree uses a replacement sampling method to obtain the training data and samples the features in a certain proportion. It can process high-dimensional data without feature selection. For unbalanced data sets, errors can be balanced; however, random forests may overfit on noisy data sets [42].

GBDT is also an integrated model based on decision trees [43]. Unlike random forest, which uses bagging to randomly select samples, GBDT uses the boosting method; it uses a serial training method to add the results of weak classifiers to obtain the prediction value. When training the next weak classifier, it fits the residual between the predicted value of the previous round of classifiers and the true value to improve the classification result.

Adaptive boosting is an integrated learning model that combines multiple weak classifiers into a strong classifier [44]. It can increase the weight of a sample that was misclassified by the previous weak classifier adaptively and train the next weak classifier. It has a better classification effect than a single decision tree [44].

### Training and Evaluation

We divided 50,216 samples into training and test sets at a ratio of 7:3. The size of the training set was 35,151 samples, and the size of the test set was 15,065 samples. To tune the parameters, we used the grid search method. Specifically, we estimated the range of several important parameters in the model (such as the threshold of variance in feature selection, the number of weak classifiers, the depth of the tree, the minimal number of sample partitions, and the learning rate), and set a step size to obtain all the possible values of these parameters. The parameter combination that obtained the best model result was selected. In addition, we also used 10-fold cross-validation to improve the robustness of the model. Normalized confusion matrix, accuracy, macro-averaged precision (macro-P), macro-averaged recall (macro-R), and macro-averaged F1 score (macro-F1) were used to evaluate models. Multimedia Appendix 1 lists the evaluation criteria formulas.

### Feature Importance Evaluation

In order to understand which features have a more important impact in the classification process, we calculated the importance value of each feature. The classification models we used were all based on tree structures, and the model of tree structures has natural advantages over other classification models in terms of interpretability. There are 2 ways to calculate the importance of features: Variable importance and Gini importance. Here, we used Gini importance to calculate the importance of features.

Gini importance is the degree to which the Gini index of a branch node formed by *M* is calculated for a feature *M* [45]. For the entire model, the average value of the Gini index of the feature on all trees is calculated. In the classification process based on tree structures, the faster the Gini index declines after a node splits, the greater the influence of the feature value represented by the split node on the classification result. The formula for Gini importance is shown as below.

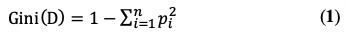

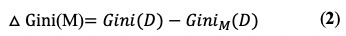

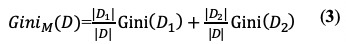







where *D* represents the entire data set, and *p_i_* represents the probability of occurrence of each class. △Gini(*M*) represents the decrease of impurity when adding the feature *M*. *D*_1_ and *D*_2_ represent the data set divided by feature *M*. The greater the value of △Gini(*M*), the higher the feature importance.

## Results

### Data Analysis

**Figure 2:**
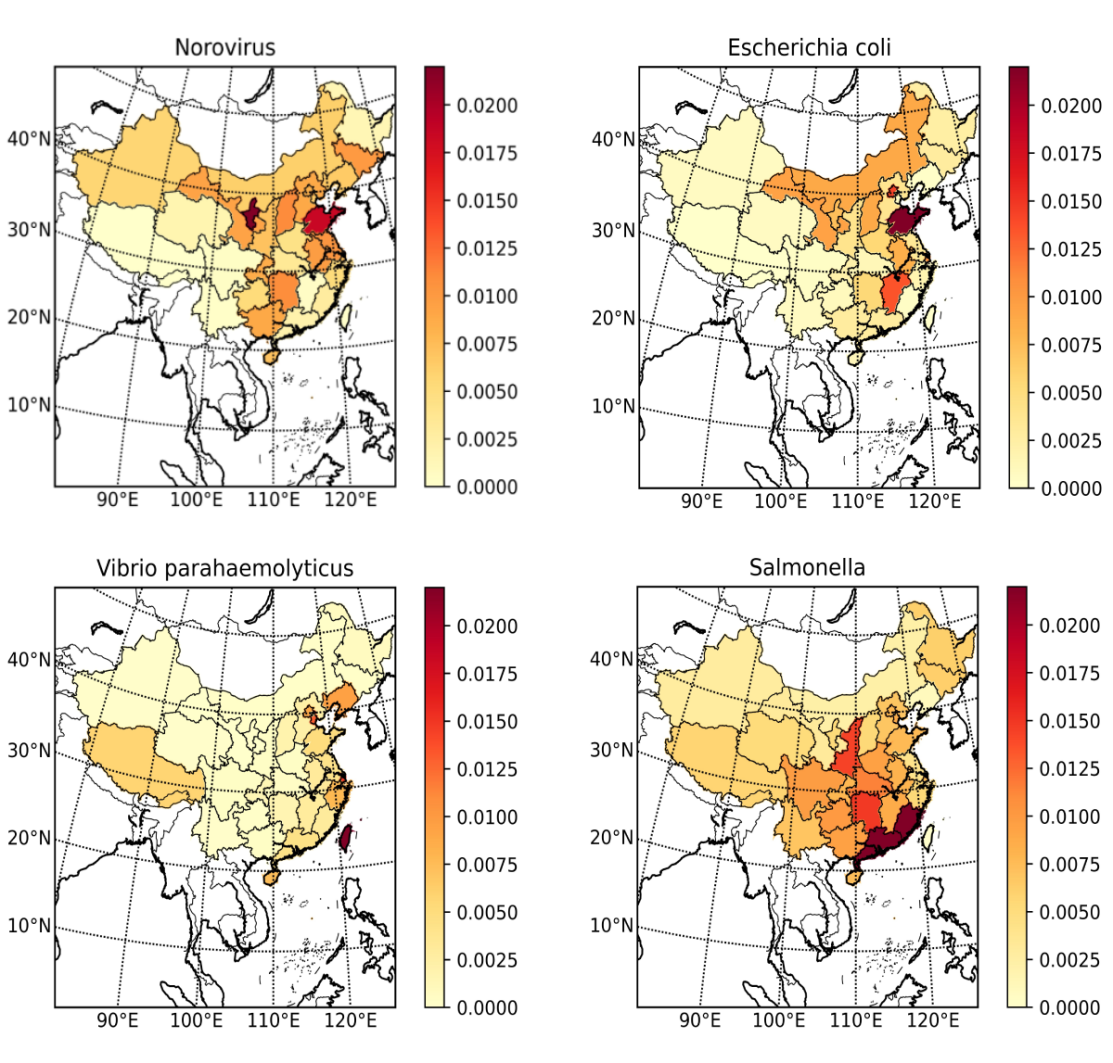
The geographic distribution of the detection rates of pathogens.

**Figure 3:**
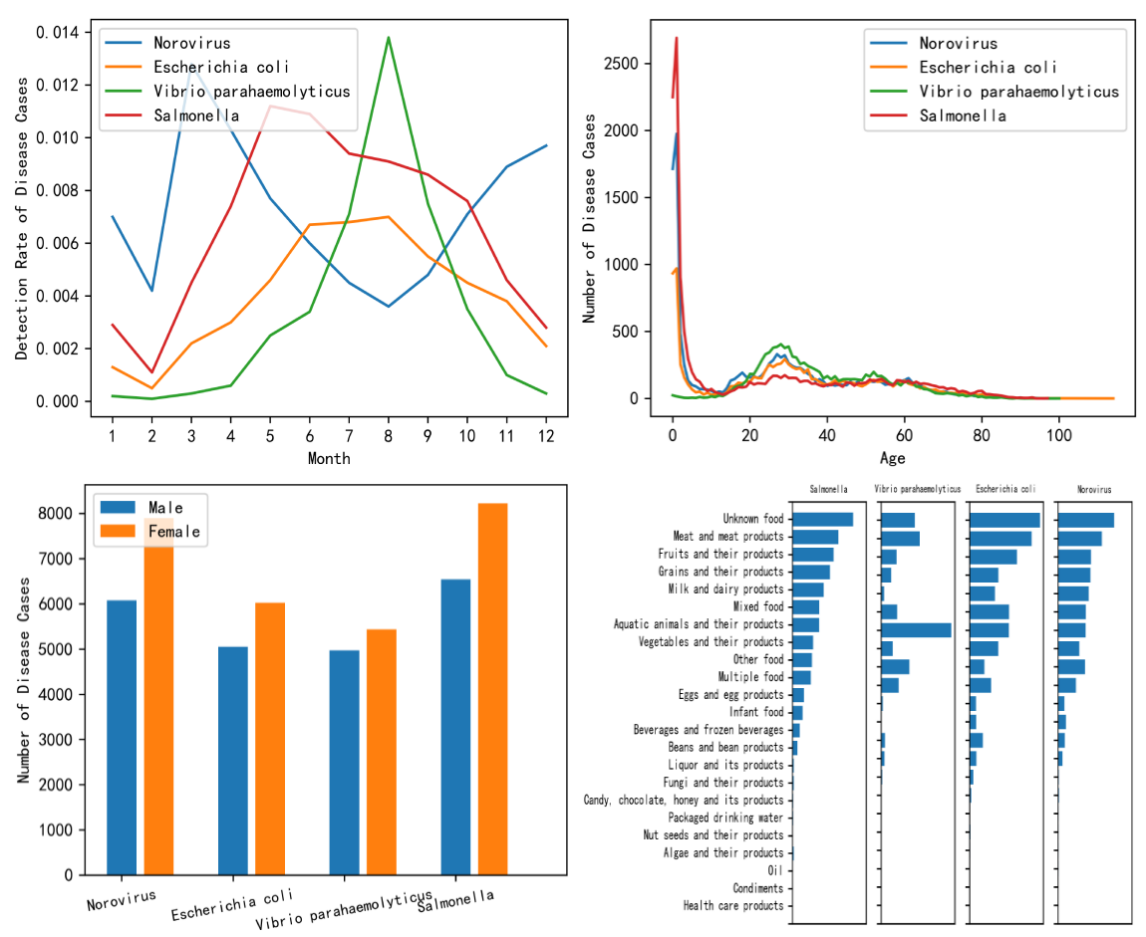
The distribution chart of the features of foodborne diseases. The upper left is the distribution of the detection rate of the pathogens by time; the upper right is the distribution of the pathogens by patient age; the bottom left is the distribution of the pathogens by patient gender; and the bottom right is the distribution of the pathogens by food type.

Through the geographical distribution of the detection rate of pathogens (Figure 2), it can be seen that the geographical distribution of the detection rate of different pathogens is somewhat distinguishable. According to the detection rate of 4 pathogens in different months as shown in the upper left of Figure 3, it can be seen that there are some differences among the 4 pathogens in seasons or months. For example, *V parahaemolyticus* occurs more frequently in summer, while *Norovirus* occurs more frequently in autumn and winter. Therefore, we can consider month as the time feature in data preprocessing. Through the distribution of age of patients of 4 pathogens (the upper right of Figure 3), the distribution trends of *E coli*, *Salmonella*, and *Norovirus* in different age groups are similar, and they were concentrated between 0 and 10 years old. Patients with *V parahaemolyticus* were between 20 and 40 years old, which was different from the other 3 pathogens. The bottom left of Figure 3 shows the gender distribution and the bottom right of Figure 3 shows the distribution of 4 pathogens in 23 food categories. These analysis results show the difference among 4 pathogens.

### Classification Results

The decision tree model's performance was worse than the those of the other 3 integrated models; its accuracy, macro-P, macro-R, and macro-F1 rate were approximately 63% (Table 4). Because the decision tree requires adjustment of fewer parameters and the model is relatively simple, we chose to use the decision tree model to perform feature selection and applied the results to the other models to reduce the number of parameters in those models that need to be adjusted. By comparing the model results under different variance thresholds, we found that increases in the word vector dimension did not greatly improve the effect of the model but increased the training time. Therefore, to balance the model effect and time cost, we finally retained a 30-dimensional word vector feature.

Each tree in the random forest model used replaceable data and feature sampling, and decision trees were parallel. The classification results were better than those for a single decision tree. After adjusting the number of decision trees, the depth of the tree, and the minimum number of split samples, the average accuracy of the random forest model was 1% higher than that of the decision tree model.

The classification results of the GBDT model were better than those of the other models. When training the GBDT model, we set the size of feature set to 0.8, which means that each single decision tree in GBDT only selects 80% of the features for training, to ensure that each training process focused on different combinations of features. After parameter tuning (weak classifier: 171; depth of the tree: 20; minimum number of sample partitions: 50), an accuracy of 69% was achieved. 

Adaptive boosting reach an accuracy of approximately 67%, only lower than that of the GBDT model.

The classification recalls of the 4 pathogens (*Norovirus*, *E coli*, *V parahaemolyticus*, *Salmonella*) were 69%, 60%, 73%, and 69%, respectively (Table 5). Among misclassified *E coli* samples, approximately 17% of the samples were misclassified as *Norovirus*, 10% of the samples were misclassified as *V parahaemolyticus*, and 13% of the samples were misclassified as *Salmonella*.

**Table 4:** The classification results of 4 classification models.

	Macro-P^a^	Macro-R^b^	Macro-F1^c^	Accuracy
Decision tree	0.62	0.63	0.63	0.63
Random forest	0.63	0.64	0.64	0.64
GBDT^d^	0.68	0.69	0.69	0.69
AdaBoost^e^	0.67	0.66	0.67	0.67

^a^Macro-P: macro-averaged precision.

^b^Macro-R: macro-averaged recall.

^c^Macro-F1: macro-averaged F1 score.

^d^GBDT: gradient boost decision tree.

^e^AdaBoost: adaptive boosting.

**Table 5:** Normalized confusion matrix of classification result in the GBDT model.

Actual	Predicted			
	*Norovirus*	*E coli*	*V parahaemolyticus*	*Salmonella*
*Norovirus*	0.69	0.13	0.06	0.13
*E coli*	0.17	0.60	0.10	0.13
*V parahaemolyticus*	0.05	0.12	0.73	0.10
*Salmonella*	0.12	0.10	0.09	0.69

### Feature Importance Evaluation

For the 4 classifiers, the top 10 important features of each classifier are shown in Table 6.

According to Table 6, we can see that the 4 classifiers have higher feature importance values in the longitude and latitude of the geographical location, the time of illness, the age of patient, the name of food, and certain symptoms (such as fever, frequency of diarrhea, frequency of vomiting). This means that these attributes have a great influence on the discrimination of pathogens. In addition, GBDT, decision tree, and AdaBoost also have relatively high importance value on diarrhea traits, and the stomachache symptom has a high impact on the classification process of the AdaBoost model and the random forest model. In the food types, aquatic animals and their products had a high impact on the classification process using decision tree or random forest. Combined with the previous exploratory analysis of data distribution, we can find that the attributes with large differences in data distribution have larger attribute importance values too.

**Table 6:** The top 10 important features in the 4 classifiers.

Importance rank	Decision tree	Random forest	GBDT^a^	AdaBoost^b^
1	Latitude	Sick time	Latitude	Latitude
2	Sick time	Latitude	Longitude	Longitude
3	Longitude	Longitude	Sick time	Sick time
4	Age of patients	Age of patients	Diarrhea frequency	Age of patients
5	Fever	Fever	Age of Patients	Diarrhea Frequency
6	Vomiting frequency	Aquatic animals and their products	Diarrhea traits	Food name
7	Diarrhea frequency	Vomiting frequency	Food name	Diarrhea frequency
8	Food name	Diarrhea frequency	Vomiting frequency	Diarrhea traits
9	Aquatic animals and their products	Food Name	Fever	Fever
10	Diarrhea traits	Stomachache	Gender of patients	Stomachache

^a^GBDT: gradient boost decision tree.

^b^AdaBoost: adaptive boosting.

## Discussion

### Principal Results

We used foodborne disease case data to visually analyze several features of foodborne diseases, and we found that the analysis results were consistent with those of previous studies in some aspects. For example, *Norovirus* occurs more frequently in autumn and winter [46], and distribution trends of patients’ age of *E coli*, *Salmonella*, and *Norovirus* are concentrated between 0 and 10 years old, which is consistent with a study result that young children are more susceptible to foodborne diseases [5]. Besides, for the 4 foodborne pathogens, there were differences in geographical, time of illness, patients’ age, patients’ gender, and exposed food categories distribution.

Of the 4 machine learning methods that we used, the best-performing classification model was the GBDT model with a classification accuracy up to 69% with the optimal parameters being 171 weak classifiers, depth of the tree—20, and minimum number of sample partitions—50, the dimension of word vector of food name—30. The classification recall of *V parahaemolyticus* was the highest, reaching almost 73%, while for *E coli,* it was only 60%. The model was most likely to mistake *Norovirus* for *E coli*. Based on this result, it can be reasonably inferred that the *V parahaemolyticus* is different from the other 3 pathogens (with respect to disease case information), and *E coli* and *Norovirus* may have similarities in distribution areas, time of illness, disease symptoms, and patient information.

We found that the 4 classifiers have higher feature importance values for time of illness, geographical longitude and latitude, and patient age. The optimal GBDT model had higher feature importance values in terms of diarrhea frequency, food name, and diarrhea traits. This result is consistent with the previous data analysis to a certain extent, such as the distribution of the 4 pathogens in geographical space, time, and patient age is quite different, so it further proves that our method is reasonable.

### Primary Contribution

Supervised learning was conducted to extract distinguishable features of different pathogens, then we compared the results of multiple experiments to obtain the optimal classification model for predicting possible pathogens for cases with unknown pathogens. The classification accuracy of the optimal model for *Salmonella*, *Norovirus*, *E coli*, and *V parahaemolyticus* can reach 69%. The model also has good scores on other evaluation indicators. Our contributions can be summarized as below:

1. We proposed a machine learning model that can automatically predict pathogens without laboratory testing. This model can potentially reduce the burden of demand for domain knowledge and technical equipment.
2. We conducted a formal analysis of the relationship between pathogens and several features of disease cases. This approach help find some distinguishable features of different pathogens.
3. Our approach can assist doctors to quickly identify the pathogens of foodborne diseases, especially if there is no sufficient test equipment and budget. This can help doctors give specific medical treatment for foodborne diseases caused by different pathogens and provide support for more accurate diseases burden estimation. It may also lead to a more accurate foodborne disease outbreak prediction.

### Limitations

This study had certain limitations. First, it should be noted that the disease case data come from a surveillance platform, and results are, therefore, influenced by the quality of the surveillance platform data—though the data were confirmed cases from hospitals or the CDC, and thus very reliable, the scope was limited. Many people may choose to buy nonprescription drugs rather than go to the hospital for treatment when their illness is not as severe; therefore, the number of disease cases collected in the surveillance platform may be lower than the actual value [14]. To solve this problem, aggregating other data sources, such as social network data or search engine data, is a useful solution. Second, a large number of patients were between 0 and 10 years old. Although some studies have shown that the burden of disease caused by foodborne disease is higher in young children [46], it has not excluded that children have a higher probability of visiting a doctor after illness than adults. Third, in the geographical distribution of pathogens, there were some differences for the 4 pathogens, but distribution may be affected by population size and economic status. For example, the population and economic conditions in the eastern part of China are better than those in western part, thus the incidence rate in the east may be higher than that in the west.

### Conclusions

We presented a machine learning–based classification method for pathogens of foodborne diseases using the case data of foodborne diseases in the National Foodborne Disease Surveillance Reporting System. Our optimal model achieved a 69% classification accuracy rate on *Salmonella*, *Norovirus*, *E coli*, and *V parahaemolyticus*. Pathogens are the main cause of foodborne diseases, research on pathogens is essential for foodborne diseases; however, due to the time and technical limitations, pathogen detection is generally performed in only a few cases, causing difficulty for identification and diagnosis of diseases. We proposed a classification method that can predict pathogens of diseases without laboratory testing. Although this method cannot replace traditional laboratory testing, it can be used to assist traditional identification with little time cost and equipment requirements. This method can help to quickly identify and diagnose foodborne disease and offer some guidance for specific medical treatments for foodborne diseases caused by different pathogens. In addition, it can also provide some support for improving accuracy rate in further foodborne diseases burden estimation and outbreak prediction.

In the future, we plan to compare our results with data from the foodborne disease outbreak surveillance system for optimization guidance, and we will try to add other domain knowledge or refer to other data sources to get more reliable results. In addition, we will carry out disease outbreak prediction.

## Appendix

Multimedia Appendix 1 Evaluation criteria.

## Abbreviations

CDC: Centers for Disease Control
GBDT: gradient boost decision tree
Macro-F1: macro-averaged F1 score
Macro-P: macro-averaged precision
Macro-R: macro-averaged recall
WHO: World Health Organization

## References

[1] Dodd C, Aldsworth T, Stein R, Cliver D, Riemann H, Jones JL (2017). Foodborne Diseases.

[2] Bean H, Griffin P, Goulding JS (1990). Foodborne disease outbreaks, 5-year summary, 1983-1987. Journal of Food Protection.

[3] Oliver SP (2019). Foodborne pathogens and disease special issue on the national and international PulseNet network. Foodborne Pathog Dis.

[4] Liu J, Bai L, Li W, Han H, Fu P, Ma X, Bi Z, Yang X, Zhang X, Zhen S, Deng X, Liu X, Guo Y (2018). Trends of foodborne diseases in China: lessons from laboratory-based surveillance since 2011. Front Med.

[5] Kirk MD, Pires SM, Black RE, Caipo M, Crump JA, Devleesschauwer B, Döpfer Dörte, Fazil A, Fischer-Walker CL, Hald T, Hall AJ, Keddy KH, Lake RJ, Lanata CF, Torgerson PR, Havelaar AH, Angulo FJ (2015). World Health Organization estimates of the global and regional disease burden of 22 foodborne bacterial, protozoal, and viral diseases, 2010: a data synthesis. PLoS Med.

[6] Centers for Disease Control and Prevention (1997). Foodborne Diseases Active Surveillance Network, 1996. MMWR Morb Mortal Wkly Rep.

[7] Foodborne Disease Monitoring and Reporting System. National Center for Food Safety Risk Assessment.

[8] Oldroyd RA, Morris MA, Birkin M (2018). Identifying methods for monitoring foodborne illness: review of existing public health surveillance techniques. JMIR Public Health Surveill.

[9] Scallan E, Hoekstra RM, Angulo FJ, Tauxe RV, Widdowson M, Roy SL, Jones JL, Griffin PM (2011). Foodborne illness acquired in the United States—major pathogens. Emerg Infect Dis.

[10] Mandal P, Biswas A, Choi K, Pal U (2011). Methods for rapid detection of foodborne pathogens: an overview. American Journal of Food Technology.

[11] Law JW, Ab Mutalib N, Chan K, Lee L (2014). Rapid methods for the detection of foodborne bacterial pathogens: principles, applications, advantages and limitations. Front Microbiol.

[12] Naravaneni R, Jamil K (2005). Rapid detection of food-borne pathogens by using molecular techniques. J Med Microbiol.

[13] Pan W, Zhao J, Chen Q (2015). Classification of foodborne pathogens using near infrared (NIR) laser scatter imaging system with multivariate calibration. Sci Rep.

[14] Flint JA, Van Duynhoven Yvonne T, Angulo FJ, DeLong SM, Braun P, Kirk M, Scallan E, Fitzgerald M, Adak GK, Sockett P, Ellis A, Hall G, Gargouri N, Walke H, Braam P (2005). Estimating the burden of acute gastroenteritis, foodborne disease, and pathogens commonly transmitted by food: an international review. Clin Infect Dis.

[15] Kuehn BM (2014). Agencies use social media to track foodborne illness. JAMA.

[16] Sadilek A, Kautz H, DiPrete L, Labus B, Portman E, Teitel J, Silenzio V (2017). Deploying Nemesis: preventing foodborne illness by data mining social media. AIMag.

[17] Effland T, Lawson A, Balter S, Devinney Katelynn, Reddy Vasudha, Waechter HaeNa, Gravano Luis, Hsu Daniel (2018). Discovering foodborne illness in online restaurant reviews. J Am Med Inform Assoc.

[18] Nogueira M, Greis N (2011). Rule-based complex event processing for food safety and public health.

[19] Bean N, Goulding J, Lao C (1997). Surveillance for foodborne disease outbreaks–United States, 1988-1992. J Food Prot.

[20] Lynch M, Painter J, Woodruff R, Braden C, Centers for Disease Control and Prevention (2006). Surveillance for foodborne-disease outbreaks--United States, 1998-2002. MMWR Surveill Summ.

[21] Swaminathan B, Barrett TJ, Hunter SB, Tauxe RV (2001). PulseNet: the molecular subtyping network for foodborne bacterial disease surveillance, United States. Emerg Infect Dis.

[22] Allard MW, Strain E, Melka D, Bunning K, Musser SM, Brown EW, Timme R (2016). Practical value of food pathogen traceability through building a whole-genome sequencing network and database. J Clin Microbiol.

[23] Hendriksen RS, Vieira AR, Karlsmose S, Lo Fo Wong DM, Jensen AB, Wegener HC, Aarestrup FM (2011). Global monitoring of Salmonella serovar distribution from the World Health Organization Global Foodborne Infections Network Country Data Bank: results of quality assured laboratories from 2001 to 2007. Foodborne Pathog Dis.

[24] Liu X, Chen Y, Wang X, Ji R (2004). [Foodborne disease outbreaks in China from 1992 to 2001 national foodborne disease surveillance system]. Wei Sheng Yan Jiu.

[25] Liu X, Chen Y, Fan Y, Wang M (2006). [Foodborne diseases occurred in 2003--report of the National Foodborne Diseases Surveillance System, China]. Wei Sheng Yan Jiu.

[26] Chen Y, Guo Y, Wang Z, Liu X, Liu H, Dai Y, Tang Z, Wen J (2010). [Foodborne disease outbreaks in 2006 report of the National Foodborne Disease Surveillance Network, China]. Wei Sheng Yan Jiu.

[27] Wallace D, Van Gilder T, Shallow S, Fiorentino T, Segler S D, Smith K E, Shiferaw B, Etzel R, Garthright W E, Angulo F J (2000). Incidence of foodborne illnesses reported by the foodborne diseases active surveillance network (FoodNet)-1997. FoodNet Working Group. J Food Prot.

[28] Dewey-Mattia D, Manikonda K, Hall AJ, Wise ME, Crowe SJ (2018). Surveillance for foodborne disease outbreaks - United States, 2009-2015. MMWR Surveill Summ.

[29] Xiao X, Ge Y, Guo Y (2015). Automated detection for probable homologous foodborne disease outbreaks.

[30] Jordan MI, Mitchell TM (2015). Machine learning: Trends, perspectives, and prospects. Science.

[31] Aramaki E, Maskawa S, Morita M (2011). Twitter Catches the Flu: Detecting Influenza Epidemics Using Twitter. Proceedings of the Conference on Empirical Methods in Natural Language Processing.

[32] Friedman JH, Meulman JJ (2003). Multiple additive regression trees with application in epidemiology. Stat Med.

[33] Teyhouee A, McPhee-Knowles S, Waldner C (2017). Prospective detection of foodborne illness outbreaks using machine learning approaches.

[34] Vilne B, Meistere I, Grantiņa-Ieviņa L, Ķibilds J (2019). Machine learning approaches for epidemiological investigations of food-borne disease outbreaks. Front Microbiol.

[35] Thakur M, Olafsson S, Lee J, Hurburgh CR (2010). Data mining for recognizing patterns in foodborne disease outbreaks. Journal of Food Engineering.

[36] D'Souza Rennie M, Becker NG, Hall G, Moodie KBA (2004). Does ambient temperature affect foodborne disease?. Epidemiology.

[37] Mikolov T, Sutskever I, Chen K, Corrado GS, Dean J (2013). Distributed representations of words and phrases and their compositionality. Advances in Neural Information Processing Systems.

[38] Li S, Zhao Z, Hu R (2018). Analogical reasoning on Chinese morphological and semantic relations. Proceedings of the 56th Annual Meeting of the Association for Computational Linguistics.

[39] Maaten L, Hinton G (2008). Visualizing Data using t-SNE. J Mach Learn Res.

[40] Varoquaux G, Buitinck L, Louppe G, Grisel O, Pedregosa F, Mueller A (2015). Scikit-learn. GetMobile: Mobile Comp and Comm.

[41] Safavian S, Landgrebe D (1991). A survey of decision tree classifier methodology. IEEE Trans Syst Man Cybern.

[42] Ho T (1995). Random decision forests. Proceedings of 3rd International Conference on Document Analysis and Recognition.

[43] Friedman JH (2001). Greedy function approximation: a gradient boosting machine. Ann. Statist.

[44] Freund Y, Schapire RE (1997). A decision-theoretic generalization of on-line learning and an application to boosting. Journal of Computer and System Sciences.

[45] Gordon Ad, Breiman L, Friedman Jh, Olshen Ra, Stone Cj (1984). Classification and regression trees. Biometrics.

[46] Ahmed SM, Lopman BA, Levy K (2013). A systematic review and meta-analysis of the global seasonality of norovirus. PLoS One.

