# Supplementary material for: Machine Learning Prediction of Foodborne Disease Pathogens: Algorithm Development and Validation Study
Source: JMIR Med Inform. 2021 Jan 26;9(1):e24924. doi: 10.2196/24924 (PMC7872834; doi:10.2196/24924)
Supplement: Multimedia Appendix 1 [file medinform_v9i1e24924_app1.docx]

**Multimedia Appendix 1**. The formulas of 4 evaluation criteria.

| Evaluation criterion | Formula |
| --- | --- |
| Accuracy | Accuracy=$\frac{TP+TN}{TP+FN+FP+TN}$ |
| Macro-P | Precision=$\frac{TP}{TP+FP}$ Macro-P=$\frac{1}{n}\sum_{1}^{n} P_{i}$ |
| Macro-R | Recall=$\frac{TP}{TP+FN}$ Macro-R=$\frac{1}{n}\sum_{1}^{n} R_{i}$ |
| Macro-F1 | F1=$\frac{2\times Precision\times Recall}{Precision+Recall}$ Macro-F1= $\frac{1}{n}\sum_{1}^{n} F_{i}$ |
